# Mediator role of psychological resilience between post-traumatic stress symptoms and burnout in academicians affected by the earthquakes in Türkiye

**DOI:** 10.3389/fpsyg.2024.1468655

**Published:** 2024-11-27

**Authors:** Ayşegül Yetkin Tekin

**Affiliations:** Psychological Counseling and Guidance Department, Faculty of Education, Adıyaman University, Adıyaman, Türkiye

**Keywords:** academician, burnout, earthquake, psychological resilience, traumatic stress

## Abstract

**Purpose:**

This study aims to investigate the mediator role of psychological resilience in the relationship between post-traumatic stress and burnout symptoms in academicians affected by two earthquakes that occurred on February 6, 2023, affecting 11 provinces of Türkiye.

**Methods:**

The research sample consisted of 175 academicians affected by these earthquakes. Each academician completed an online survey consisting of a sociodemographic form, the PTSD Checklist for DSM-5 (PCL-5), the Brief Psychological Resilience Scale, and the Burnout Syndrome Inventory-Short Version.

**Results:**

Negative relationships were found between the post-traumatic stress symptom and burnout levels of academicians and their psychological resilience levels. Psychological resilience had a partial mediating role in the relationships of post-traumatic stress symptoms (reexperiencing, avoidance, and hyperarousal) and burnout.

**Conclusion:**

It can be said that higher post-traumatic stress symptoms’ severity is associated with higher levels of burnout, and psychological resilience has a mediator role in the relationship between post-traumatic stress and burnout in earthquake-affected academicians.

## 1 Introduction

On February 6, 2023, two devastating earthquakes with a magnitude of 7.7 at 4:17 and 7.6 at 13:24 occurred, and these earthquakes affected 11 provinces in the south and southeast parts of Türkiye (Aydan and Ulusay, 2023). According to the official data of the Republic of Türkiye, approximately 50 thousand people lost their lives in these earthquakes, in which approximately 15 million people were affected^1^. The earthquake survivors also faced severe social and professional problems, and their housing and working conditions were negatively impacted. Although a long time has passed since these earthquakes, some survivors still live in containers and prefabricated buildings (Tatar et al., 2024). These earthquakes caused severe problems and disruptions in the education system in the affected cities. Many schools and universities were seriously damaged in the earthquakes, and in addition to accommodation problems, education-related difficulties arose for students and teachers (Yıldız et al., 2023; Güneser and Saygili, 2024).

Post-traumatic stress disorder (PTSD) is one of the most common mental disorders in earthquake survivors (Cénat et al., 2020). According to the findings of a recently published meta-analysis, the prevalence of PTSD in earthquake victims can vary between 4.1 and 67% (Tang et al., 2017). It has been reported that the prevalence of PTSD in adult earthquake survivors affected by the earthquakes that occurred on February 6, 2023, in Türkiye is over 50% (Han Alpay et al., 2024; İlhan et al., 2023). PTSD may also be observed in earthquake survivors for long after the earthquake (Fong et al., 2022; Karamustafalıoğlu et al., 2023). Many individual, demographic, and psychosocial risk factors for PTSD have been reported in earthquake survivors (Sirotich and Camisasca, 2024; Lu et al., 2021). It has been reported that critical social factors such as losing a loved one in the earthquake, damage of the house, and poor psychosocial support are essential risk factors, especially for long-term PTSD (Tanrikulu and Kayaoglu, 2024; Yılmaz et al., 2024; Alipour and Ahmadi, 2020).

One important psychological disturbance in earthquake survivors is burnout (Hendriati et al., 2024; Mattei et al., 2017). Especially face-to-face workers, such as healthcare workers and teachers, have been shown to suffer from burnout symptoms after earthquakes (Mattei et al., 2017; O’Toole and Friesen, 2016; Johal et al., 2016). Burnout is a syndrome characterized by physical and mental exhaustion, fatigue, and depersonalization, especially seen in work groups that provide direct face-to-face service to people (Maslach and Leiter, 2016). Long-term or chronic stress, increased workload, and negative changes in working conditions can also cause burnout (Edú-Valsania et al., 2022). PTSD can cause burnout due to prolonged stress response and increased sensitivity to stress, deterioration in social relationships and behaviors, and negative emotions (Wang et al., 2022; Restauri and Sheridan, 2020).

Psychological resilience is defined as all the skills to adapt to stressful life conditions (Sisto et al., 2019). Psychological resilience is one of the critical factors affecting coping with the effects of adverse life events and post-traumatic growth (Masten et al., 2021). Many factors, such as personality traits, social support, childhood experiences, coping strategies, and education level, affect psychological resilience (Mlinac and Schwabenbauer, 2018). It is known that a higher level of psychological resilience has a protective effect against the emergence of mental disorders in individuals affected by stressful life events (Brooks et al., 2020). For example, it has been indicated that a high psychological resilience level in earthquake survivors is a protective factor against the emergence of PTSD (Chen et al., 2022; Xi et al., 2020).

On the other hand, it has been reported that individuals with high levels of psychological resilience experience less burnout (Khaksar et al., 2019). It has been revealed that a higher psychological resilience level plays a protective role against burnout symptoms after stressful life events (Gonzalez et al., 2019; Treglown et al., 2016). The relationship between psychological resilience and both PTSD and burnout has led to the investigation of its mediating role between these two variables (Liu et al., 2023; Jeong and Shin, 2023; Jeong and Song, 2022). For example, a partial mediating effect of psychological resilience has been reported between secondary traumatic stress and burnout in intensive care nurses (Jeong and Shin, 2023). Again, a partial mediating effect of psychological resilience has been shown between the severity of post-traumatic stress and emotional exhaustion levels of nurses during the COVID-19 pandemic (Jeong and Song, 2022).

**Figure 1:**
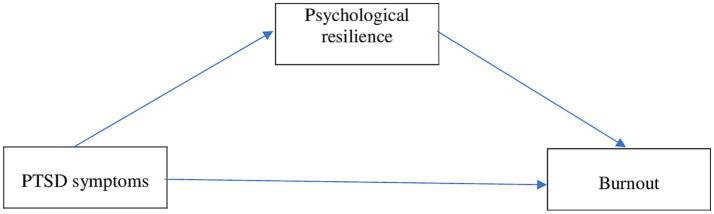
Theoretical model of the research.

According to the information mentioned above, it can be said that post-traumatic stress and burnout symptoms are common in earthquake survivors, and post-traumatic stress symptoms may be associated with more severe burnout symptoms in earthquake survivors. Moreover, while there is a negative relationship between the severity of post-traumatic stress symptoms and psychological resilience, psychological resilience has a protective effect against burnout symptoms. Therefore, it can be thought psychological resilience may have a mediator role that can affect the relationship between post-traumatic stress symptoms and burnout in earthquake survivors. Indeed, a mediator is defined as a variable that can change the effect of an independent variable on a dependent variable (Baron and Kenny, 1986). As summarized in Figure 1, the main hypotheses of this study were: (1) there may be a positive relationship between post-traumatic stress symptoms and burnout, (2) there may be a negative relationship between psychological resilience and both post-traumatic stress and burnout, and (3) psychological resilience may have a mediator effect between post-traumatic stress and burnout academicians who were affected by the earthquakes on February 6, 2023, and continue to work in the same province.

## 2 Method

### 2.1 Participants

The study had a cross-sectional and descriptive nature. The research sample consisted of 175 academicians working at Adıyaman University and affected by the earthquakes on February 6, 2023. The forms used in the research were prepared as an electronic survey and sent to the academicians’ corporate e-mail addresses with the permission of Adıyaman University Rectorate. Responses were received from 189 academicians between May 1 and July 4, 2024. The responses of 14 academicians were removed from the data set due to missing data.

All stages of the study were approved by Adıyaman University Social and Human Sciences Ethics Committee (Approval Date: 24.04.2024, and Approval Number: 44). All academicians participating in the research marked the confirmation link on the informed consent page located at the first page of the electronic survey. Those who did not mark the link on the informed consent page could not access the pages containing other materials.

### 2.2 Materials

#### 2.2.1 Socidemographic form

The form included some demographic features of the academicians, such as age, gender, marital status, income level, loss of relative during the earthquakes, house damage level during the earthquakes, current place of residence (home, container or prefabricated structure, etc.), and living situation with family.

#### 2.2.2 Turkish version of the PTSD checklist for DSM-5 (PCL-5)

It is a five-point Likert-type scale consisting of 20 items prepared according to DSM-5 PTSD diagnostic criteria (Blevins et al., 2015). It is used to evaluate the severity of traumatic stress symptoms (re-experiencing, avoidance, negative alterations, hyperarousal). Each item is scored between 0 and 4, and the total score that can be obtained from the scale is between 0 and 80. Higher scores on the scale indicate more severe traumatic stress symptoms. Turkish validity and reliability study was conducted by Boysan et al. (2017). Cronbach’s alpha coefficients in the Turkish form are 0.84 for re-experiencing, 0.78 for avoidance, 0.87 for negative alterations, 0.83 for hyperarousal, and 0.94 for the entire scale.

#### 2.2.3 Brief psychological resilience scale

It is a five-point Likert-type self-report scale consisting of 6 items, developed to measure psychological resilience against stressful life events (Smith et al., 2008). Each item is scored between 1 and 5 points. A high score from the scale indicates high psychological resilience. The Turkish validity and reliability study was conducted by Doğan (2015), and the Cronbach alpha coefficient for the Turkish form is 0.83.

#### 2.2.4 Burnout syndrome inventory-short version

It was developed by Pines (2005), and its Turkish validity and reliability study was conducted by Tümkaya et al. (2009). It is a seven-point Likert-type self-report scale consisting of 10 items. Increasing total scores on the scale indicate higher levels of burnout. The internal consistency coefficient for the Turkish form is 0.91.

### 2.3 Statistical analysis

SPSS 25.0 package program and Jamovi 2.4.14 statistical program were used in the statistical analysis of the data. Descriptive characteristics of the participants were given as mean, standard deviation, number and percentage. Whether continuous variables conformed to normal distribution or not was interpreted according to the Kolmogorov–Smirnov test. The relationship between the academicians’ traumatic stress symptom severity, burnout severity, and psychological resilience levels was examined with Spearman correlation analysis. Harman single factor test was used to assess common method variance bias, and all self-report scale scores used in the study were subjected to non-rotation factor analysis. When the number of factors is limited to 1, the total variance explained is 41.4%, and if the rate was lower than 50%, it was accepted there was no common method variance error in the study. Before the mediation analysis, multicollinearity between the independent variables was checked with Variance Inflation Factor (VIF) values. Since VIF values were below 5, it was accepted that there was no multicollinearity between the independent variables (O’brien, 2007). Mediational models were created in order to investigate the role of psychological resilience between post-traumatic stress symptoms (reexperiencing, avoidance, negative alterations, and hyperarousal) and burnout. Sobel test was performed in order to interpret the significance of mediational models. In all statistical analyses, the significance level was accepted as *p* < 0.05.

## 3 Results

50.3% (*n* = 88) of the participants were male and 49.7% (*n* = 87) were female. The average age of the participants was 41.8 (SD = 7.9, Range = 24–61). 12.6% (*n* = 22) of the participants were professors, 32% (*n* = 56) were associate professors, 41.7% (*n* = 73) were assistant professors, and 13.7% (*n* = 24) were research assistants. 31.4% (*n* = 55) of the participants lost at least one relative in the earthquakes. The sociodemographic characteristics of the participants are shown in Table 1.

**Table 1:** Sociodemographic features of the earthquake-affected academicians.

Variable		Mean ± Standart deviation or number (Percentage)
Age		41.8 ± 7.9
Gender	Female	87 (49.7)
	Male	88 (50.3)
Academic title	Professor	22 (12.6)
	Associate Professor	56 (32)
	Assistant Professor	73 (41.7)
	Research Assistant	24 (13.7)
Marital status	Married	116 (66.3)
	Single	47 (26.9)
	Other	12 (6.9)
Income level	High	41 (23.4)
	Medium	108 (61.7)
	Low	26 (14.9)
Loss of relative during the earthquakes	Yes	55 (31.4)
Damage level of home	Severe	48 (27.4)
	Medium	47 (26.9)
	Slight	62 (35.4)
	Undamaged	18 (10.3)
Current housing condition	Home	126 (72)
	Container or prefabricated structure	39 (22.3)
	Other (guesthouse, hotel)	10 (5.7)
Living with family	Yes	121 (69.1)

There were negative relationships between psychological resilience and re-experiencing, avoidance, negative alterations, and hyperarousal in the participants (*r* = −0.39, *r* = −0.35, *r* = −0.36, and *r* = −0.38, respectively). There were positive relationships between burnout levels and re-experiencing, avoidance, negative changes, and hyperarousal levels in the participants (*r* = 0.50, *r* = 0.46, *r* = 0.41, and *r* = 0.51, respectively). A significant negative relationship was found between the participants’ psychological resilience levels and their burnout levels (*r* = −0.46) (Table 2).

**Table 2:** Correlations between levels of traumatic stress symptoms, psychological resilience, and burnout in earthquake-affected academicians.

Scale	M ± SD	Range	1	2	3	4	5	6
1. Re-experiencing	10.4 ± 3.8	0–20	1					
2. Avoidance	4.2 ± 1.9	0–8	0.88**	1				
3. Negative alterations	15.3 ± 4.7	0–28	0.73**	0.79**	1			
4. Hyperarousal	13.3 ± 5.2	0–24	0.78**	0.78**	0.81**	1		
5. Psychological resilience	17.4 ± 3.6	6–30	−0.37**	−0.33**	−0.35**	−0.33**	1	
6. Burnout	36.9 ± 11.7	10–70	0.50**	0.45**	0.40**	0.49**	−0.43**	1

M, mean; SD, standard deviation, **p < 0.01.

**Figure 2:**
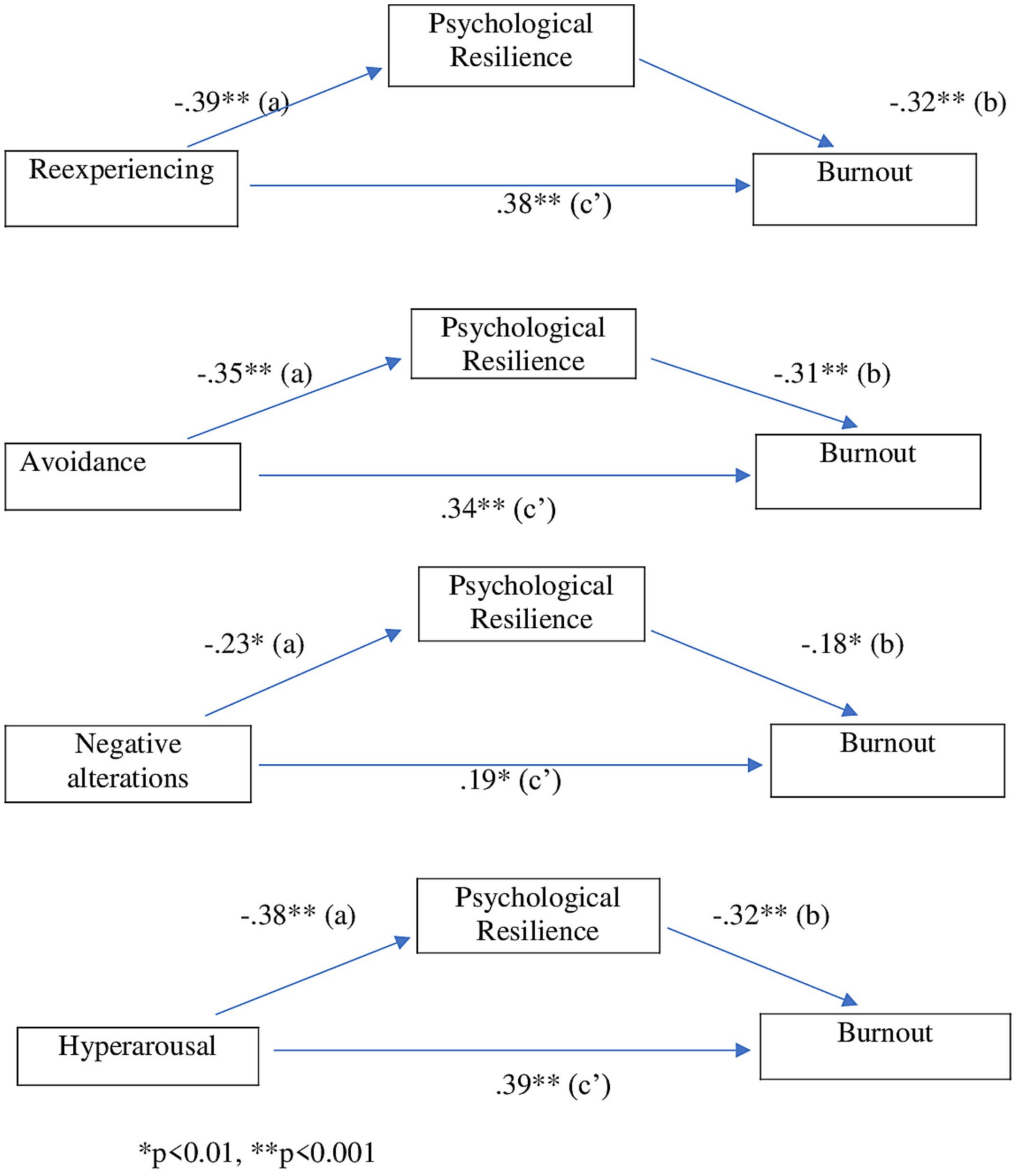
The mediator role of psychological resilience between post-traumatic stress symptoms and burnout in earthquake-affected academicians (standardized path *β* coefficients are given).

Figure 2 and Table 3 showed the mediator role of psychological resilience in the relationship between post-traumatic stress symptoms (re-experiencing, avoidance, negative alterations, and hyperarousal) and burnout in earthquake-affected academicians. Accordingly, psychological resilience had partial mediator effects in the relationships of burnout and reexperiencing (*β* = 0.38, *p* < 0.001), avoidance (*β* = 0.34, *p* < 0.001), negative alterations (*β* = 0.19, *p* = 0.017) and hyperarousal (*β* = 0.39, *p* < 0.001). However, according to the Sobel test results, the mediator effect of psychological resilience was not found to be valid in the relationship of negative alterations and burnout (*Z* = 1.31, *p* = 0.0059).

**Table 3:** Mediator effect of psychological resilience between the relationships of post-traumatic stress symptoms and burnout in earthquake-affected academicians.

Mediating models	Direct effect			Indirect effect			Total effect			Sobel test	
	SE	*t*	*p*	SE	*t*	*p*	SE	*t*	*p*	*Z*	*p*
Reexperiencing-PR-burnout	0.19	−3.65	<0.001	0.10	−2.55	0.009	0.19	−5.64	<0.001	2.39	0.029
Avoidance-PR-burnout	0.44	−2.97	0.002	0.23	−2.54	0.01	0.44	−4.77	<0.001	2.06	0.037
Negative alterations-PR-burnout	0.14	−2.09	0.019	0.07	−1.61	0.033	0.14	−3.25	0.017	1.31	0.059
Hyperarousal-PR-burnout	0.16	−3.84	<0.001	0.08	−2.73	0.004	0.16	−5.82	<0.001	2.88	0.019

## 4 Discussion

This study aims to examine the relationship between traumatic stress, psychological resilience, and burnout and the mediating role of psychological resilience in the relationships between post-traumatic stress symptoms and burnout in academicians affected by two severe earthquakes that occurred approximately 9 h apart on February 6, 2023 and affected 11 provinces in southeastern Türkiye. The findings of this study have shown that there are negative relationships between the psychological resilience of earthquake-affected academicians and their post-traumatic stress symptoms and burnout levels. Additionally, mediational models have indicated that psychological resilience has a partial mediator effect in the relationship of reexperiencing, avoidance, and hyperarousal and burnout in earthquake-affected academicians.

The findings of many studies have shown that psychological resilience has a protective effect against the negative psychological consequences of traumatic life events (Overstreet et al., 2021; Reyes et al., 2019; Thompson et al., 2018). Studies conducted on earthquake survivors have shown that post-traumatic stress symptoms are less likely to occur in individuals with high levels of psychological resilience (Chen et al., 2022; Ikizer et al., 2016; Heetkamp and De Terte, 2015). The findings of this study have also shown that a higher level of psychological resilience is associated with lower levels of traumatic stress symptoms in earthquake-affected academicians. It can be said that the findings of our study are compatible with the findings of previous studies. It has been reported that the protective effect of psychological resilience against traumatic stress is related to many regulatory factors such as trauma-related education, social support, and functional coping strategies (Brooks et al., 2020).

The findings of this study have indicated that increased psychological resilience levels in earthquake-affected academicians are associated with lower burnout levels. Many previously published studies have already demonstrated the negative relationship between psychological resilience and burnout (Smith and Emerson, 2021; West et al., 2020; Ríos-Risquez et al., 2018; McCain et al., 2018; Chaukos et al., 2017). It has also been reported that high psychological resilience plays a vital role in coping with the psychological effects of traumatic life events and burnout. Sharifian et al. (2023) have reported that high psychological resilience was associated with low burnout symptoms in war-affected Syrian teachers. Although the relationship between psychological resilience and burnout has not been investigated directly in earthquake survivors, it can be said that we obtained findings in our study that are compatible with the findings of studies conducted with other samples.

The results of the present study have shown that psychological resilience has a partial mediator role in the relationships between post-traumatic stress symptoms (reexperiencing, avoidance, and hyperarousal) and burnout in earthquake-affected academicians. Although the mediating role of psychological resilience in the relationship between post-traumatic stress and burnout in earthquake survivors has not been investigated, this role has been reported in samples exposed to other types of trauma in a small number of studies (Liu et al., 2023; Jeong and Shin, 2023; Jeong and Song, 2022; Huang et al., 2021). While the partial mediator effect of psychological resilience between post-traumatic stress and burnout in nurses during the COVID-19 pandemic has been reported, the findings of another study have indicated the partial mediator role of psychological resilience between secondary traumatic stress symptoms and burnout in nurses working in the intensive care unit (Jeong and Shin, 2023; Jeong and Song, 2022). It has been determined that avoidant coping and problem-solving have a mediating role between post-traumatic stress symptoms and burnout in firefighters (Huang et al., 2021). Both the findings of the present study and the findings of previous studies conducted in different samples indicate that psychological resilience has a positive effect on burnout in those with post-traumatic stress symptoms. Namely, it can be said that psychological resilience reduces the impact of post-traumatic stress symptoms on burnout in individuals exposed to traumatic life events.

This study has some limitations. The cross-sectional nature of the study makes it difficult to state a definitive relationship between variables. The fact that the data obtained was obtained only from self-report scales shows a need for more objective data obtained through clinically oriented interviews. The fact that concepts such as psychological resilience and burnout can be affected by many variables has made it difficult to interpret the effect of confounding factors. Finally, the relatively small sample size of the research and the fact that the sample included earthquake-affected academicians in only one of the 11 provinces affected by the earthquakes make it difficult to generalize the findings. Future studies with larger samples may help to interpret the role of confounding factors in the relationship between post-traumatic stress, burnout symptoms, and psychological resilience in earthquake-affected academicians.

## 5 Conclusion

To conclude, traumatic stress and burnout levels in earthquake-affected academicians are related to psychological resilience. As the level of traumatic stress increases in earthquake-affected academicians, their burnout levels also increase. Determining the psychological and social needs of earthquake-affected academicians may be useful in preventing burnout in the post-traumatic process. Psychosocial intervention programs that can help earthquake-affected academicians cope with traumatic stress can help them cope with burnout.

## Data Availability

The data presented in the study are included in the article/supplementary material, further inquiries can be directed to the corresponding author/s.

## Footnotes

^1^ https://www.sbb.gov.tr/wp-content/uploads/2023/03/Turkiye-Recovery-and-Reconstruction-Assessment.pdf
